# Transcriptomic and metabolomic profiling of ionic liquid stimuli unveils enhanced secondary metabolism in *Aspergillus nidulans*

**DOI:** 10.1186/s12864-016-2577-6

**Published:** 2016-04-12

**Authors:** Paula C. Alves, Diego O. Hartmann, Oscar Núñez, Isabel Martins, Teresa L. Gomes, Helga Garcia, Maria Teresa Galceran, Richard Hampson, Jörg D. Becker, Cristina Silva Pereira

**Affiliations:** Instituto de Tecnologia Química e Biológica António Xavier, Universidade Nova de Lisboa, Av. da República, 2780-157 Oeiras, Portugal; Department of Analytical Chemistry, University of Barcelona, Diagonal 645, E-08028 Barcelona, Spain; Serra Hunter Fellow, Generalitat de Catalunya, Barcelona, Spain; Thelial Technologies S.A., Parque Tecnológico de Cantanhede, Nucleo 04 Lote 3, 3060-197 Cantanhede, Portugal; Instituto Gulbenkian de Ciência, Rua da Quinta Grande 6, 2780-156 Oeiras, Portugal

**Keywords:** *Aspergillus nidulans*, Monodictyphenone, Orsellinic acid, Secondary metabolism, Ionic liquids, Metabolomics, Transcriptomics

## Abstract

**Background:**

The inherent potential of filamentous fungi, especially of Ascomycota, for producing diverse bioactive metabolites remains largely silent under standard laboratory culture conditions. Innumerable strategies have been described to trigger their production, one of the simplest being manipulation of the growth media composition. Supplementing media with ionic liquids surprisingly enhanced the diversity of extracellular metabolites generated by penicillia. This finding led us to evaluate the impact of ionic liquids’ stimuli on the fungal metabolism in *Aspergillus nidulans* and how it reflects on the biosynthesis of secondary metabolites (SMs).

**Results:**

Whole transcriptional profiling showed that exposure to 0.7 M cholinium chloride or 1-ethyl-3-methylimidazolium chloride dramatically affected expression of genes encoding both primary and secondary metabolism. Both ionic liquids apparently induced stress responses and detoxification mechanisms but response profiles to each stimulus were unique. Primary metabolism was up-regulated by choline, but down-regulated by 1-ethyl-3-methylimidazolium chloride; both stimulated production of acetyl-CoA (key precursor to numerous SMs) and non proteinogenic amino acids (building blocks of bioactive classes of SMs). In total, twenty one of the sixty six described backbone genes underwent up-regulation. Accordingly, differential analysis of the fungal metabolome showed that supplementing growth media with ionic liquids resulted in *ca.* 40 differentially accumulated ion masses compared to control conditions. In particular, it stimulated production of monodictyphenone and orsellinic acid, otherwise cryptic. Expression levels of genes encoding corresponding polyketide biosynthetic enzymes (i.e. backbone genes) increased compared to control conditions. The corresponding metabolite extracts showed increased cell polarity modulation potential in an ex vivo whole tissue assay (*Thelial Li*ve *T*argeted *E*pithelia; *the*LiTE™).

**Conclusions:**

Ionic liquids, a diverse class of chemicals composed solely of ions, can provide an unexpected means to further resolve the diversity of natural compounds, guiding discovery of fungal metabolites with clinical potential.

**Electronic supplementary material:**

The online version of this article (doi:10.1186/s12864-016-2577-6) contains supplementary material, which is available to authorized users.

## Background

Multiple and diverse fungal secondary metabolites (SMs) are already in clinical usage, e.g. the antibiotic penicillin and the antitumor terrequinone A [1]. The inherent SM biosynthetic capacity of fungi remains largely unseen because the majority of these pathways are largely silent (cryptic) under culture conditions used in the laboratory [2]. The presence of various SM backbone genes (encoding non-ribosomal peptide synthases, polyketide synthases, hybrid enzymes, prenyltransferases or terpene cyclases) in fungal genomes hints at the presence of an array of uncharacterised SMs. For example, model fungal species *Aspergillus nidulans* has sixty six predicted backbone genes [3]; approximately one third of these clusters have been linked to the full range of produced SMs, including monodictyphenone and prenyl xanthones [4, 5], asperfuranone [6], emericellamides [7], aspyridone A/B [8], asperthecin [9], terrequinone A [1, 10], aspernidine A [11], sterigmatocystin [12], penicillin [13], nidulanin A [14], microperfuranone [15], cichorine [16], orsellinic acid and F9775 A/B [17], austinol and dehydroaustinol [18] and aspercryptin [19].

Closing the gap between genetic potential and the observed diversity of fungal SMs produced constitutes a major challenge [20], further complicated by low production titers and the need for specific stimuli to trigger synthesis [21]. Several strategies have been described to stimulate production of particular SMs; some require prior knowledge of genomic sequences, relying on manipulation of targeted genes encoding components of either secondary metabolism [22] (e.g. aspoquinolones A–D [23]) or regulatory pathways (e.g. monodictyphenone [24] and asperthecin [9]). Other approaches may be applied also in less well characterised strains, such as co-cultivation methods (e.g. culturing together *Emericella* spp. and *Salinispora arenicola* triggers production of two cyclic depsipeptides [25]) or modification of the growth media composition (e.g. addition of sodium citrate or suberoylanilide hydroxamic acid increased the production of terrein in *A. terreus* [26] and nygerone A in *A. niger*, respectively [27]). We have surprisingly observed that supplementation of growth media with ionic liquids can significantly increase diversity of compounds in the metabolic footprint of penicillia [28]. Ionic liquids comprise a diverse class of chemicals, composed solely by ions and are commonly classified as alternative green solvents (despite many having pronounced toxic effects) [29]. They are also referred to as task-designed solvents, because their properties can be tuned through simple modification of the structure of either ion [30]. More than 10^9^ different formulations are theoretically possible, with thousands already available commercially. Recently we analysed the major cellular responses of *A. nidulans* to either cholinium chloride or 1-ethyl-3-methylimidazolium chloride exposure [31]. Selected chemical stimuli are representative of the most studied families of ionic liquids and represent opposite ends of the spectrum regarding toxicity and recalcitrance. Both compounds increase numerous mycelial stress-responsive proteins (e.g. drug transporter proteins) and induce particular developmental changes and production of certain osmolytes [31]. Extracellular compound diversity was apparently greater in *A. nidulans* grown in media supplemented with either ionic liquid than in control cultures. In summary the possibility of ionic liquids being able to activate cryptic SM biosynthetic pathways in fungi deserves further investigation. Here we analyse how cholinium chloride and 1-ethyl-3-methylimidazolium chloride impact on both primary and secondary metabolism in *A. nidulans*. Differential analyses of the fungal metabolome were combined with targeted gene expression analysis and transcriptional profiling (custom Affymetrix microarray [32]). Data highlight ionic liquid’s capacity to impact both on primary and secondary metabolism, stimulating SM biosynthesis (e.g. the cryptic SM monodictyphenone). The diversity of differentially formed metabolites apparently comprised also unknown compounds with cell polarity modulation potential (*the*LiTE™). This study sheds first light on the vast potential of ionic liquids to reveal the diversity of natural compound biosynthesis potential in fungi.

## Methods

### Chemicals

All standard chemicals (toluhydroquinone, orcinol, epoxysuccinic acid, phenoxyacetic acid, 2,5-dihydroxybenzoic acid (gentisic acid), fusaric acid, 3-(3,4-dihydroxyphenyl)-2-propenoic acid (caffeic acid), propyl-3,4,5-trihydroxybenzoate, jasmonic acid, sterigmatocystin, penicillin G, physcion and riboflavin) and chromatographic solvents were of highest analytical grade and purchased from either Sigma Aldrich or Fisher Scientific, except ethyl acetate (Acros Organics), orsellinic acid (Alfa Aesar) and chrysophanol (Acros Organics). Water was obtained from a Milli-Q system (Millipore). Cholinium chloride (>98 %, Sigma Aldrich), hereafter referred to solely as choline, and 1-ethyl-3-methylimidazolium chloride ([C_2_mim]Cl, >98 %, Iolitec) were dried *in vacuo* (40–70 °C, 24–48 h, *ca.* 0.01 mbar) prior to use. Monodictyphenone was kindly provided by Prof. Thomas J. Simpson (University of Bristol, UK) [33].

### Fungal strain

*Aspergillus nidulans* strain FGSC A4 was cultivated on dichloran-glycerol (DG18) agar (Oxoid), and suspensions of fungal conidia, prepared as previously described [31], were stored at −80 °C in cryoprotective solution containing 0.85 % w/v NaCl and 10 % v/v glycerol.

### Culture condition

Fungal cultures (5 mL or 50 mL) were initiated from conidia (10^5^ conidia *per* mL) in a 0.1 % glucose mineral growth media [31] alone (control) or containing 0.7 M [C_2_mim]Cl or choline (dosage equivalent to 50 % of the minimal inhibitory concentration of [C_2_mim]Cl [31]). Liquid cultures (triplicates) were incubated in the dark at 27 °C with orbital agitation (90 rpm), for defined periods of time (2, 5, 7, 10 or 15 days). At the end of incubation, fungal mycelia (mostly submerged) were recovered by filtration (glass fibre pre-filters) and both mycelia and filtrate were immediately frozen in liquid nitrogen and stored at −80 °C, until further analysis.

### Microscopic analysis

Mycelia were recovered from fungal cultures after fifteen days of incubation (50 mL; inoculated and incubated as described above, triplicate samples), mounted on glass slides and stained with lactophenol blue to enhance contrast. Visualisation of cultures was performed using a DM5500 B microscope (Leica) with 40× or 63× magnification objectives and images were captured with a DFC420 C camera (Leica).

### RNA isolation and cDNA synthesis

Total RNA was isolated from mycelia (previously ground to a powder using mortar and pestle in liquid nitrogen) using the RNeasy Plant Mini Kit (QIAGEN) and further purified following standard ethanol precipitation. Quantity and quality of RNA was determined using a NanoDrop 1000 Spectrophotometer (Thermo Scientific) and RNA integrity assessed by using an Agilent 2100 Bioanalyser with a RNA 6000 Nano Assay (Agilent Technologies). cRNA was fragmented and biotinylated according to GeneChip 3’ IVT Express Kit protocols. Briefly, 100 ng total RNA were used for cDNA synthesis, which was in vitro transcribed to generate labelled cRNA. After purification and fragmentation, the size distribution of cRNA and fragmented cRNA was assessed using an Agilent 2100 Bioanalyzer with an RNA 6000 Nano Assay.

### DNA microarray processing

The custom DNA microarray FungiANC (Affymetrix) was used in this study [32]. The chip contains a total of 20,012 transcripts derived from the genetic information of *A. nidulans* and *Neurospora crassa* (Broad Institute Database, www.broadinstitute.org) and is based on a Perfect Match-only design with 11 μm feature size. Each transcript is represented by 11 oligonucleotides of 25-*mer* (detailed description in Additional file 1). The array was processed following Affymetrix GeneChip protocols, in biological triplicates. A total of 200 μl hybridization mixture containing 10 μg fragmented cRNA was hybridized to arrays for 16 h at 45 °C. Standard post-hybridization washes and double-stain protocols (FS450_0001) were used on an Affymetrix GeneChip Fluidics Station 450, in conjunction with the GeneChip Hybridization Wash and Stain Kit (Affymetrix). Arrays were scanned on an Affymetrix GeneChip Scanner 3000 7G. All array quality parameters were analysed by Expression Console Software (Affymetrix) for Robust Multiarray Averaging (summarised data) and confirmed to be in the recommended range. The data herein presented have been deposited in NCBI’s Gene Expression Omnibus [34] and are accessible through GEO Series accession number GSE65946 (www.ncbi.nlm.nih.gov/geo/query/acc.cgi?acc=GSE65946).

### Microarray data analysis

Microarray data analysis was performed using DNA-Chip Analyzer (dChip) software (www.dchip.org, 2010), applying a probeset mask file considering only *A. nidulans* probes (9674 transcripts). Arrays were normalised to a baseline array with median CEL intensity by applying an Invariant Set Normalization Method [35, 36]. Normalised CEL intensities of the 9 arrays were used to obtain model-based gene expression indices based on a Perfect Match-only model [35, 36]. Log2 expression data produced by dChip was imported into R v2.13.0 and differential gene expression analysed with the Bioconductor LIMMA package (www.bioconductor.org) [37]. Principal component analysis and volcano plots were obtained to validate biological replicates and visualise distribution of statistically significant data from each biological condition (Additional file 1). Differentially expressed genes (adjusted *p*-value ≤ 0.05, |FC| ≥ 1.5), identified using pair-wise comparison between each condition and control (grown on glucose) (Additional file 2), were analysed with Venn diagrams (Venny, http://bioinfogp.cnb.csic.es/tools/venny/index.html) (Additional file 1).

### Functional annotation

Annotation of all genes represented on the DNA microarray was obtained from the Broad Institute Database and the *Aspergillus* Genome Database (www.aspgd.org). Full details are given in Additional file 2. Differentially expressed genes for each condition were classified using the FungiFun web annotation tool (https://sbi.hki-jena.de/FungiFun) [38]. Significant hits (*p*-value ≤ 0.05) were defined using the identities present on the chip as background (Additional file 1).

### Quantitative real-time PCR

All quantitative real-time PCR (*q*RT-PCR) oligonucleotide pairs – based on *A. nidulans* gene sequences (*Aspergillus* Genome Database, www.aspgd.org) - were designed using the GeneFisher2 web tool (http://bibiserv.techfak.uni-bielefeld.de/genefisher2) and produced by Thermo Fisher Scientific (Additional file 1). *q*RT-PCR analyses were performed in a CFX96 Thermal Cycler (Bio-Rad), using the SsoFast EvaGreen Supermix (Bio-Rad), 250 nM of each oligonucleotide and cDNA template equivalent to 1 ng total RNA, in a final volume of 10 μl *per* well (three technical and three biological replicates). PCR conditions were: enzyme activation at 95 °C for 30 s; 40 cycles of denaturation at 95 °C for 10 s and annealing/extension at 59 °C for 30 s; and melting curve obtained from 65 °C to 95 °C, consisting of 0.5 °C increments every 5 s. Data analysis was performed using the CFX Manager Software v3.0 (Bio-Rad). Expression of each gene was calculated relative to control. Expression of all target genes was normalised to the expression of the histone H3 gene, used as internal control.

### Metabolite extraction and analyses

Lyophilised culture filtrates were homogenised in Milli-Q water and extracted three times with ethyl acetate (1:1), dried under soft nitrogen flow and resuspended in a minimal volume of methanol. Preliminary mass spectrometry analyses of the extracts showed much higher abundance of differential *m/z* in negative mode compared to positive mode (data now shown). For that reason, ultra-high performance liquid chromatography-electrospray ionisation-high resolution mass spectrometry (UHPLC-ESI-HRMS) analyses of metabolite extracts were performed in negative mode using a Q-Exactive Orbitrap MS system (ThermoFisher Scientific) equipped with a heated electrospray ionisation source (HESI-II) [39]. Chromatographic separation was carried out in an UHPLC system (Accela; ThermoFisher Scientific) using an Ascentix Express C18 (150 × 2.1 mm, 2.7 μm particle size) column from Supelco (USA). The mobile phase, at a flow rate of 300 μL/min, consisted of a solution of 0.1 % formic acid (solvent A) and a solution of acetonitrile containing 0.1 % formic acid (solvent B), set as follows: 10 % B in 1 min, followed by a liner gradient of 10–95 % B in 4.7 min, 1.3 min to reach 100 % B, 3 min of 100 % B, 0.5 min to return to the initial conditions, and 5.5 min to re-equilibrate the column. HESI-II was operated in negative ionisation mode. Nitrogen was used as a sheath gas, sweep gas and auxiliary gas at flow rates of 60, 0 and 10 a.u. (arbitrary units), respectively. Heater temperature was set at 350 °C. Capillary temperature was set at 320 °C and electrospray voltage at − 2.5 kV. A S-Lens RF level of 50 V was used. Q-Exactive Orbitrap MS system was tuned and calibrated using ThermoFisher calibration solution once a week. The HRMS instrument was operated in full MS scan with a *m/z* range from 50 to 600, and the mass resolution tuned into 70,000 full width half maximum (FWHM) at *m/z* 200, with an automatic gain control (AGC) target (the number of ions to fill C-Trap) of 5.0E5 with a maximum injection time (IT) of 200 ms. The full MS scan was followed by a data-dependent scan operated in All Ion Fragmentation (AIF) mode with a fragmentation energy applied of 30 eV into the high-energy collision dissociation (HCD) cell. At this stage, mass resolution was set at 17,500 FWHM at *m/z* 200, AGC target at 5.0E5, maximum IT at 200 ms, and the scan range also from *m/z* 50 to 600. MS data were processed by ExactFinder™ v2.0 software (Thermo Fisher) by applying a user target database list, comprising nearly one thousand four hundred SMs previously described in Ascomycota (Additional file 3). Parameters including retention time, accurate mass errors and isotopic pattern matches were used in preliminary manual compound identification. Analytical standards of monodictyphenone, chrysophanol, orsellinic acid, toluhydroquinone, orcinol, epoxysuccinic acid, phenoxyacetic acid, 2,5-dihydroxybenzoic acid (gentisic acid), fusaric acid, 3-(3,4-dihydroxyphenyl)-2-propenoic acid (caffeic acid), propyl-3,4,5-trihydroxybenzoate, jasmonic acid, sterigmatocystin, physcion and riboflavin were used for compound identity validation, applying following criteria: ΔRT ≤ 0.2 min and Δ(m/z) ≤ 5 ppm. Mycelial accumulation of betaine was quantified as previously reported [31].

### Ex vivo assay for anti-carcinoma activity

Cell polarity is central to onset and progression of diseases including carcinoma; validated assay *the*LiTE™ measures polarity modulating activity of compounds in live *Drosophila* tissues. All tests were conducted as disclosed in published US patent application 20130136694. Briefly, egg chambers are extracted from female *Drosophila* less than 7 days old and exposed to metabolites (pure compounds and both the crude metabolite extracts and their polar fractions [40]) at standardised concentrations in Schneider’s culture medium under controlled standard atmospheric conditions for up to 6 h. Egg chambers are observed using standard light and fluorescence microscopy and scored for presence/absence of polarity marker protein Par6. Each assay is done in triplicate. Controls for these assays included pure compounds: *the*-103 (a functional equivalent of aurothiomalate [41], which displays 100 % activity in *the*LiTE™), monodictyphenone, orsellinic acid and either ionic liquid, as well as the blank (0.6 % v/v DMSO) and metabolite extracts of the control cultures and the non-inoculated media.

## Results and discussion

### *Aspergillus nidulans* differential metabolic footprints under ionic liquid stimuli

**Fig. 1:**
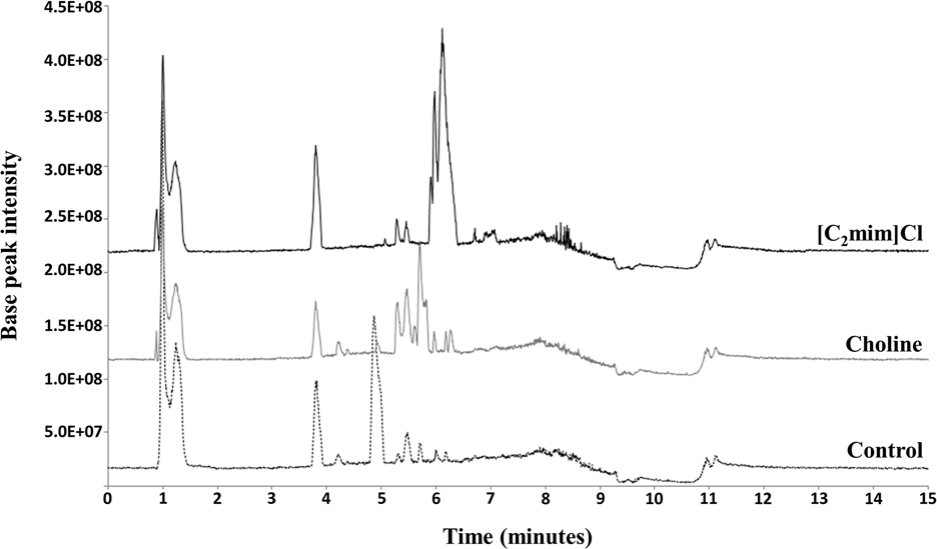
Chromatographic analyses of the metabolic footprint of *Aspergillus nidulans* under ionic liquid stimuli. The base peak intensity chromatograms of the culture extracts after fifteen days of incubation in either choline or 1-ethyl-3-methylimidazolium chloride ([C_2_mim]Cl) supplemented media revealed higher diversity of metabolites when compared to the control

The diversity of compounds in the metabolic footprint of fungi depends on growth media composition (e.g. carbon and nitrogen sources availability) [42]. Simple, systematic compositional alterations allow discovery of multiple SMs in a single producing organism [43]. This strategy, usually known as the “one strain-many compounds approach”, provides high flexibility for screening poorly characterised strains. Adding sub-lethal concentrations of an ionic liquid to the growth media of *Penicillium* spp. [28] or *A. nidulans* [31] augmented the diversity of compounds in the culture footprints. High resolution spectrometric analyses of *A. nidulans* metabolite extracts were performed to expand our initial findings. Compared to control conditions ionic liquid stimuli altered the fungal metabolic footprint, increasing the diversity of metabolites (Fig. 1). By applying a user target list of Ascomycota SMs, a list of differential ion masses (*m/z*) detected under an ionic liquid stimulus, when compared to the control, could be produced (Additional file 3). There were *ca.* 40 differential ion masses detected in either ionic liquid medium when compared to the control, with 24 and 18 specific to choline or 1-ethyl-3-methylimidazolium chloride supplemented media, respectively (Additional file 3).

Most ion masses matched compounds present in our user target list but only six compound identifications could be validated with standards (Table 1). These included four known metabolites of *Aspergillus*: orcinol [44, 45], phenoxyacetic acid [46], orsellinic acid [17] and monodictyphenone [24], as well as gentisic acid and caffeic acid which, to the best of our knowledge, are reported here for the first time in *A. nidulans*. Gentisic acid has previously been detected in *Penicillium griseofulvum* [47], whereas caffeic acid was reported only once in a fungal metabolite screening [48]. None of the remaining putative identifications could be validated with the corresponding standards, e.g. chrysophanol and sterigmatocystin. None of the ion masses identified here matched penicillin, confirming its absence.

**Table 1:** Metabolites differentially produced under ionic liquid stimuli

*m/z*	Choline	[C_2_mim]Cl	Molecular formula	Compound identification	Reference
123.0450	^a^	✓	C7H8O2	orcinol	[34, 35]
151.0402		✓	C8H8O3	phenoxyacetic acid	[36]
153.0195	✓		C7H6O4	2,5-dihydroxybenzoic acid (gentisic acid)	[38, 39]
167.0348	✓^b, c^	✓ ^b, c^	C8H8O4	orsellinic acid	[37, 39]
179.0352	✓	✓	C9H8O4	3-(3,4-dihydroxyphenyl)-2-propenoic acid (caffeic acid)	[39]
287.0566	✓^c^		C15H12O6	monodictyphenone	[14]

UHPLC-ESI-HRMS differential analyses of putative compounds (ion masses) detected in the metabolite extracts of either choline or 1-ethyl-3-methylimidazolium chloride ([C_2_mim]Cl) supplemented media, compared to the control. Compound identifications were validated by the corresponding standards

^a^a compound reported equal m/z value but was not validated as orcinol;^b^ vestigial amounts also found in the control; ^c^also detected in early time-points: monodictyphenone was detected after 5 and 10 days of incubation in choline media; and orsellinic acid was detected after 5 days of incubation in choline media and after 10 days of incubation in [C_2_mim]Cl media

**Fig. 2:**
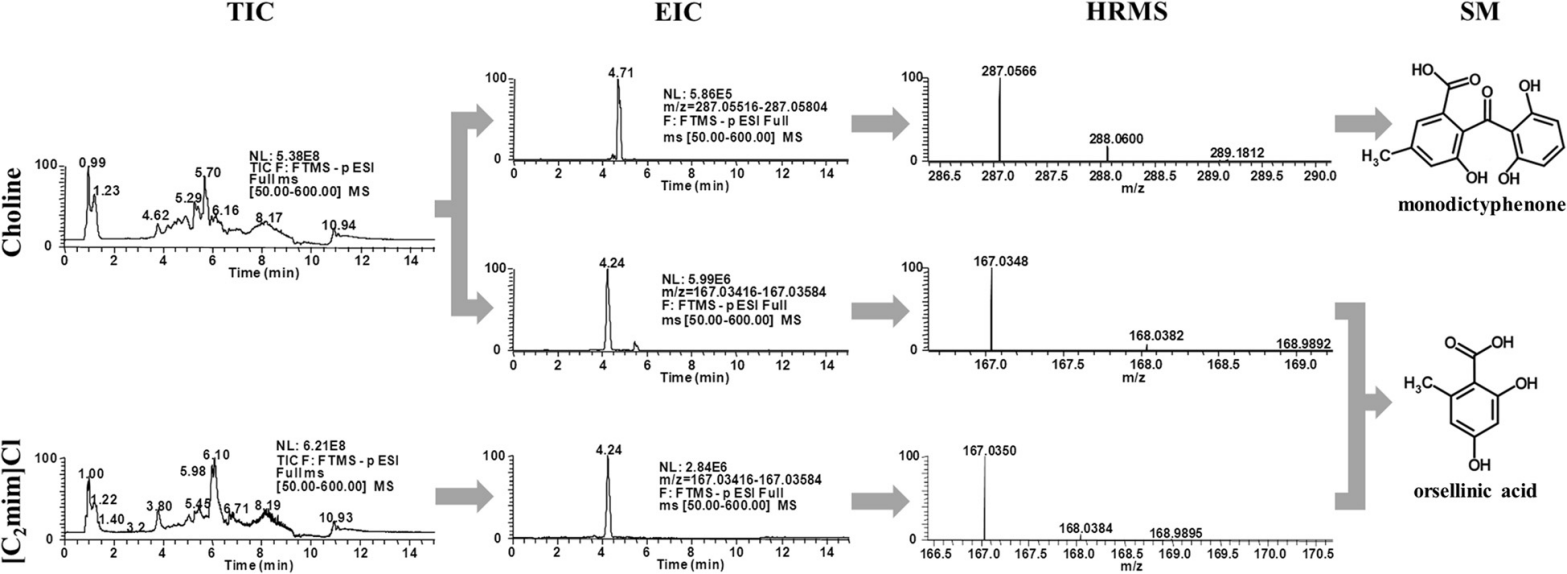
Total ion chromatogram (TIC) of *Aspergillus nidulans* metabolite extracts under ionic liquid stimuli. TIC derived from fifteen day cultures in choline or 1-ethyl-3-methylimidazolium chloride ([C_2_mim]Cl) supplemented media. Extracted ion chromatograms (EIC) and respective HRMS spectra for peaks corresponding to monodictyphenone and orsellinic acid are also presented

Among identified compounds we found two already characterised cryptic SMs (Table 1). Monodictyphenone, exclusively found in choline supplemented media, and orsellinic acid, found with both ionic liquid supplements (Fig. 2). Monodictyphenone is a product of the monodictyphenone biosynthetic pathway in *A. nidulans* [4, 5, 24, 33], initially characterised in a Δ*cclA* strain (*n.b. cclA* encodes a methyltransferase known to impact secondary metabolism) [24]. Orsellinic acid has been detected in *A. nidulans* during co-cultivation with *Streptomyces hygroscopicus* [49] and, more recently, also in sucrose supplemented media [17]. The production of the otherwise cryptic derivatives of the monodictyphenone cluster (e.g. emodin and chrysophanol), and orsellinic acid, was stimulated in *A. nidulans* grown in continuous fermentation under nutrient limited conditions [50].

### Transcriptome profiling of *A. nidulans* during exposure to ionic liquids

To shed light on the impact of ionic liquids on the *A. nidulans* transcriptome, including genes encoding components involved in secondary metabolism – either biosynthesis or regulation – whole-genome profiling analysis (FungiANC) [32] was performed. Raw signal intensities for all genes are given in Additional file 2. Principal Component Analysis showed that the biological replicates of each condition were clustered together (implying a very low degree of replicate variation in transcript levels) and dissociated from the remaining clusters (Additional file 1). Volcano plots clearly show a great number of transcripts with highly significant differential expression (Additional file 1). Pair-wise comparison was used to identify genes expressed differentially between the control and cultures grown in ionic liquid media for fifteen days (adjusted *p*-value ≤ 0.05 and |FC| ≥ 1.5). In either ionic liquid media *ca.* 35 % of transcripts (total of 9674) showed altered expression levels, but only *ca.* 6 % were altered by both (Venn diagrams in Additional file 1). Choline supplementation led to up- and down-regulation of 1252 and 811 transcripts, respectively. 1-Ethyl-3-methylimidazolium chloride supplement, up-regulated 1207 transcripts and down-regulated 1271 transcripts. According to the functional categories database of the Munich Information Center for Protein Sequences (MIPS), the differentially expressed genes were enriched in the categories metabolism (MIPS 01), energy (MIPS 02), transcription (MIPS 11) and protein synthesis (MIPS 12). In general, most of these functional categories were up-regulated in choline medium but down-regulated in 1-ethyl-3-methylimidazolium chloride medium (Additional file 1). These findings match those of our previous proteomic profiling study [31], which reflect the distinct biodegradability and toxicity of these ionic liquids.

### Ionic liquids impact on stress response and primary metabolism of *A. nidulans*

Filamentous fungi respond to very diverse environmental stresses by activating different signalling transduction cascades [51–54]. Extracellular signals are usually sensed and transmitted to response regulators [55] that also impact other processes, ranging from asexual development and cell wall integrity to fungicide sensitivity [53]. *Aspergillus nidulans* can tolerate saline concentrations considerably higher than those used here [56]. Both ionic liquids up-regulated the response regulator of the high osmolarity glycerol (HOG) pathway *– sskA* (AN7697) (Table 2), but none of the downstream elements (*pbsB*, AN0931; *hogA*, AN1017; *sskB,* AN10153) nor any transcription factors regulated by this pathway (e.g. *atfA,* AN2911 and *srrA*, AN3688) [57]. In 1-ethyl-3-methylimidazolium chloride supplemented medium, *sskA* up-regulation occurred together with genes coding for catalase A (*catA*, AN8637), glycerol-3-phosphate dehydrogenase (*gfdB*, AN6792), trehalose-6-phosphate phosphatase (*orlA*, AN3441), neutral trehalase (*treB*, AN5635) and NADP(+)-dependent glycerol dehydrogenase (*gldB*, AN5563) (Table 2), strongly suggesting the formation of stress-tolerant conidia [53, 58]. In fact, conidia formation was observed in the floating mycelia at the surface of the liquid media in both cultivation conditions.

**Table 2:** Genes of stress response differentially expressed after ionic liquid stimuli

		Transcriptional profile*		
GeneID	Gene	Choline	[C_2_mim]Cl	Description
AN7697	*sskA*	**1.81**	**1.49**	response regulator
AN0931	*pbsB*	**−1.67**	1.30	HOG signaling pathway MAPKK
AN1017	*hogA*	**−1.73**	1.01	osmotic stress-activated kinase
AN8637	*catA*	**−2.32**	**1.94**	catalase A
AN9339	*catB*	**2.66**	1.21	catalase B
AN5918	*catC*	**2.34**	**−4.09**	catalase C
AN7388	*cpeA*	**2.76**	**−2.79**	catalase D, catalase-peroxidase
AN5523	*tpsA*	**−1.85**	1.37	trehalose-6-phosphate synthase subunit 1
AN3441	*orlA*	**−1.74**	**2.62**	trehalose-6-phosphate phosphatase
AN5635	*treB*	1.39	**2.34**	neutral trehalase
AN6792	*gfdB*	**−3.56**	**2.69**	glycerol-3-phosphate dehydrogenase
AN5563	*gldB*	−1.28	**2.74**	NADP(+)-dependent glycerol dehydrogenase
*Glutathione metabolism*				
AN2846	*gpxA*	1.23	**−2.65**	glutathione peroxidase
AN4905	*gstA*	**−2.04**	−1.29	theta class glutathione S-transferase
AN3299		**3.58**	**2.11**	glutathione S-transferase
AN6158		**1.89**	**3.15**	glutathione S-transferase
AN10444	*ggtA*	**2.30**	1.48	gamma-glutamyltranspeptidase
AN5658		**2.58**	**−1.93**	gamma-glutamyltranspeptidase
AN3459		**1.98**	**−2.09**	glutamate carboxypeptidase
AN2514		−1.16	**2.81**	gamma-cysteine synthetase regulatory subunit
*Multidrug transporters*				
AN0015		**2.01**	**1.68**	ABC multidrug transporter
AN2349		**−4.55**	1.02	ABC multidrug transporter
AN6443		**2.14**	−1.28	ABC multidrug transporter
AN8150		1.48	**3.80**	ABC multidrug transporter
AN8489		**7.89**	**−1.64**	ABC multidrug transporter
AN8892		**2.10**	**73.69**	ABC multidrug transporter
AN9342		**2.11**	**2.60**	ABC multidrug transporter
AN0732		**4.78**	**2.94**	MFS multidrug transporter
AN1243		1.03	**2.17**	MFS multidrug transporter
AN1691		1.04	**2.19**	MFS multidrug transporter
AN2531		**−3.69**	**2.22**	MFS multidrug transporter
AN3301		1.36	**7.78**	MFS multidrug transporter
AN6477		**6.42**	1.48	MFS multidrug transporter
AN6942		**8.86**	**77.17**	MFS multidrug transporter
AN7295		**3.12**	1.25	MFS multidrug transporter
AN7466		**1.62**	**2.52**	MFS multidrug transporter
AN8089		**3.70**	−1.47	MFS multidrug transporter
AN8610		**3.04**	1.05	MFS multidrug transporter
AN8621		**−5.58**	**2.92**	MFS multidrug transporter
*Autolysis/autophagy*				
AN0472	*engA*	**−3.46**	**−3.39**	β-1,3-endoglucanase
AN4871	*chiB*	**−2.41**	**−5.07**	chitinase B
AN1760		**−1.81**	1.28	autophagy protein Apg12
AN3734		**−1.69**	−1.26	autophagy protein Apg9
AN5876		**−1.94**	−1.30	autophagy protein Atg22
AN6360		1.01	**1.50**	autophagy protein, ATG17 homolog
AN10213		**2.00**	**4.30**	autophagy protein Apg6

Microarray analyses (fold-change, FC) in choline or 1-ethyl-3-methylimidazolium chloride ([C2mim]Cl) supplemented media in pair-wise comparisons with the control. Values highlighted in bold are statistically significant (|FC| ≥ 1.5 and p-value ≤ 0.05)

^*^values highlighted in bold have |FC| **≥** 1.5 and *p*-value **≤** 0.05 in the microarray data

**Fig. 3:**
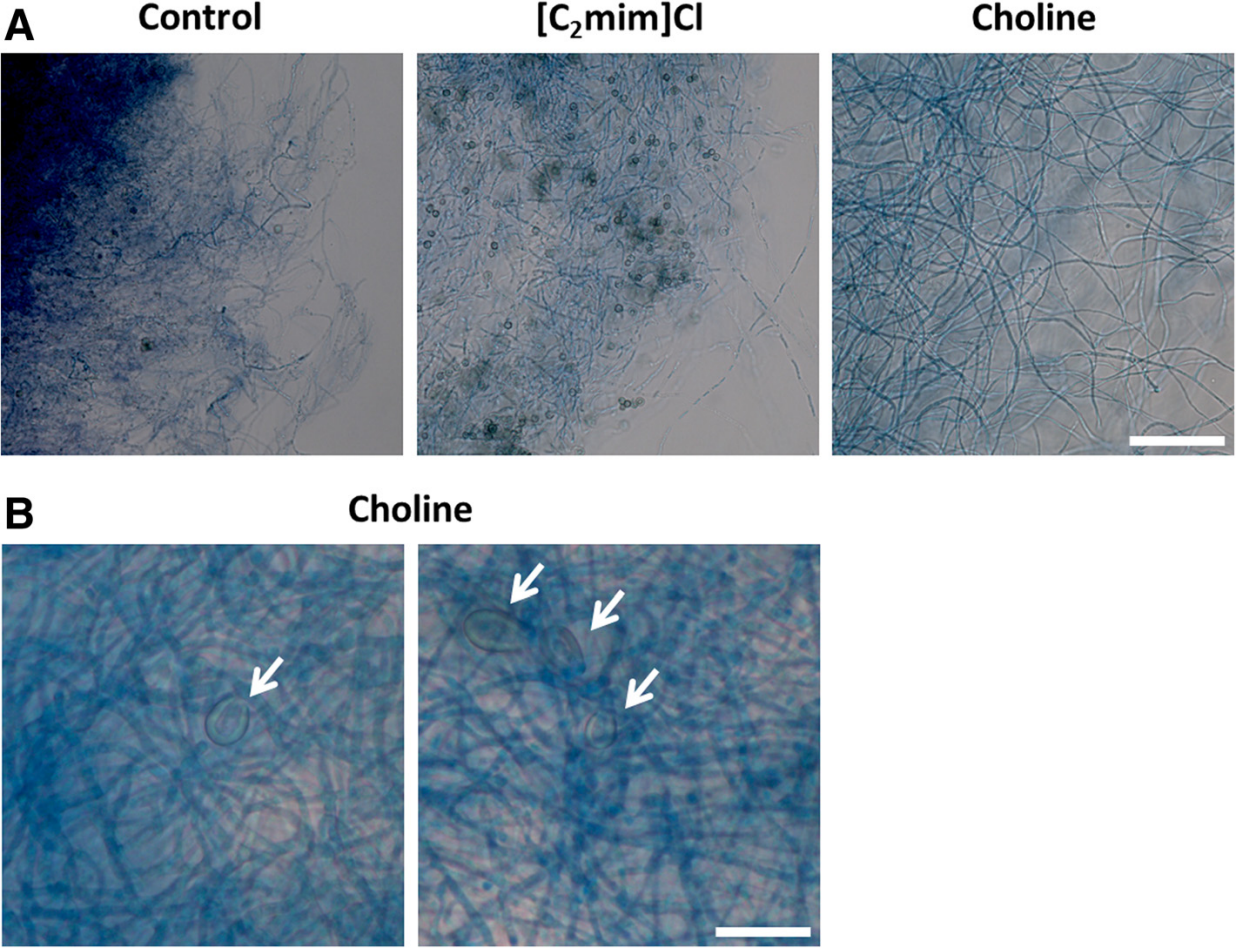
Microscopic images of *Aspergillus nidulans* mycelia under ionic liquid stimuli. Images were collected at incubation day fifteen in the control medium or in either choline or 1-ethyl-3-methylimidazolium chloride ([C mim]Cl) supplemented media (**a**). In media supplemented with choline, formation of Hülle cells could be observed (white arrows) (**b**). Scale bars: 50 μm (**a**); 30 μm (**b**)

It has previously been suggested that 1-ethyl-3-methylimidazolium chloride can induce autolysis in *A. nidulans* [31], a process of self-digestion of aged hyphae [59]. Proteome profiling showed the increase of two autolysis hallmark proteins, β-1,3-endoglucanase (EngA, AN0472) and chitinase B (ChiB, AN4871), during growth in ionic liquid supplemented media [31]. The corresponding transcripts were not found to be up-regulated here. Up-regulation of AN10213 (autophagy protein Apg6) and AN6360 (homolog of ATG17) suggests autophagy was occurring (Table 2). This process is related to nutrient recycling during starvation and has been shown to precede autolysis [60]. Microscopic analysis showed that by incubation day fifteen, mycelia and hyphae of *A. nidulans* grown in choline supplemented medium were more robust than those grown in 1-ethyl-3-methylimidazolium chloride supplemented medium, which in turn were similar to the control culture (Fig. 3a). The formation of Hülle cells was detected in the choline medium (Fig. 3b), as also seen in our previous study [31]. This is consistent with the observed up-regulation of the catalase D gene (*cpeA*, AN7388) (Table 2), induced in these specialised cells during sexual development [61].

**Fig. 4:**
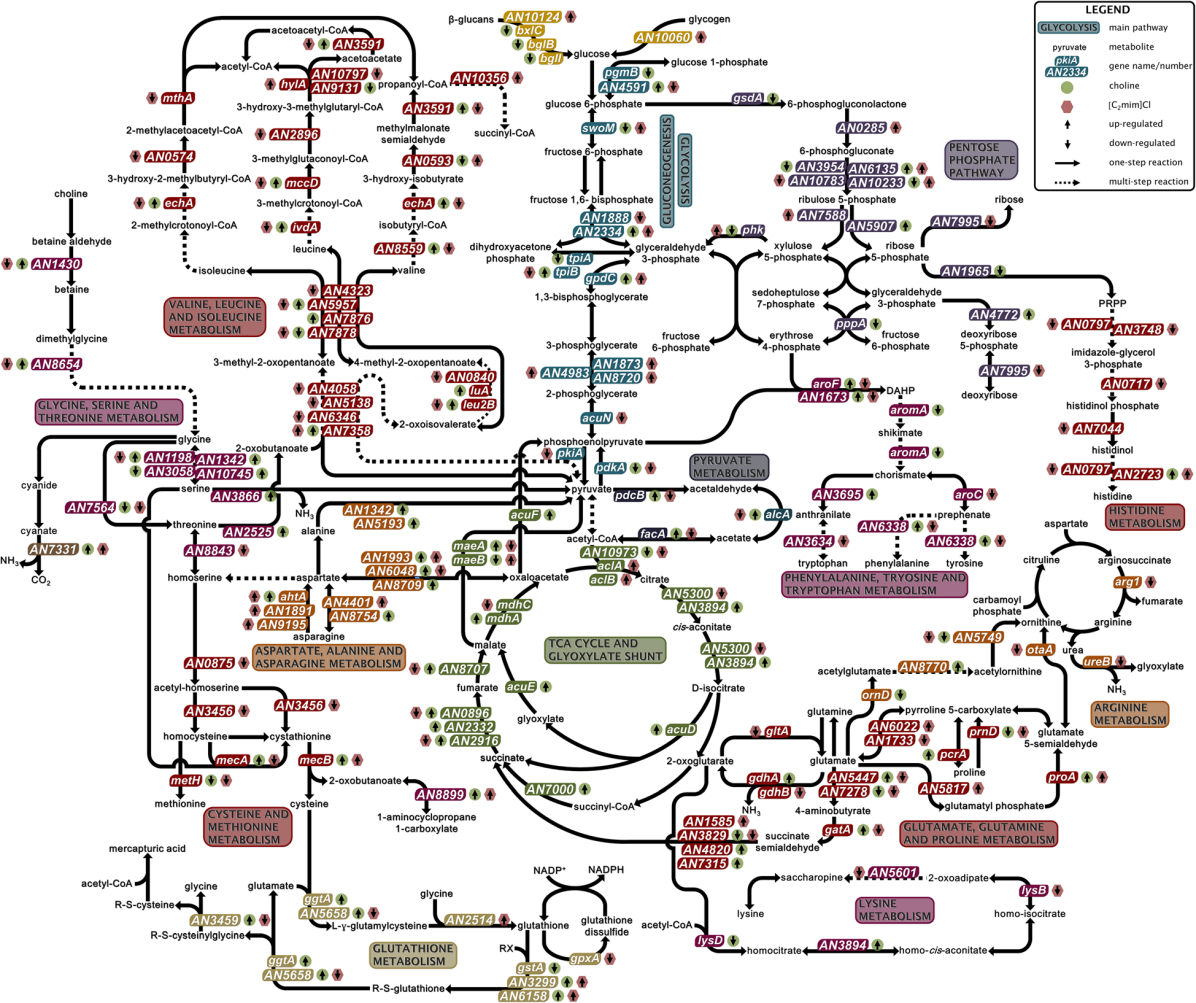
Main alterations in *Aspergillus nidulans* primary metabolism and stress response under ionic liquid stimuli. Schematic view of the main alterations in the primary metabolism (carbohydrate and amino acid metabolisms) and stress response of *Aspergillus nidulans* after fifteen days of incubation in the control medium or in either choline or 1-ethyl-3-methylimidazolium chloride ([C_2_mim]Cl) supplemented media. Many steps and compounds are omitted for simplification

*Aspergillus nidulans* is able to take up choline as a source of carbon and nitrogen [62]. In choline supplemented medium a great number of genes involved in the central carbon and amino acids metabolisms were up-regulated (Fig. 4, Additional file 1), suggesting activation of primary metabolism. Choline can be phosphorylated and incorporated into phosphatidylcholine, a principal constituent of cellular membranes [63]. Excess of phosphatidylcholine is counterbalanced by its degradation into for example 1,2-diacylglycerol, as suggested by the up-regulation of the phospholipase C gene (*plcB*, AN7691) (Additional file 2). In our previous proteomic study [31], we observed that choline was most likely taken up and metabolised via the glycine, serine and threonine metabolic pathway, and incorporated into the central carbon metabolism. The transcriptomic data reported here further confirm these observations. Up-regulation of betaine aldehyde dehydrogenase (AN1430) and dimethylglycine oxidase (AN8654) supports the hypothesis that choline enters the primary metabolism through formation of betaine aldehyde and betaine, which is further converted to glycine (Fig. 4). The three-fold accumulation of betaine in the mycelia under choline supplementation when compared to the control (39.43 ± 5.29 mg/mL and 12.25 ± 6.42 mg/mL, respectively) further supports this hypothesis. Downstream steps indicate the formation of serine from glycine (up-regulation of AN1198, AN1342 and AN10745) and its conversion into pyruvate, as evidenced by the major up-regulation of the serine dehydratase gene (AN3866), which reached almost 119-fold (Additional file 1). As a consequence of this influx of carbohydrates, most genes involved in the tricarboxylic acid (TCA) cycle and glyoxylate shunt were up-regulated (Fig. 4). Some genes involved in glycolysis/gluconeogenesis were also up-regulated (*e.g.* fructose-bisphosphate aldolase, AN2334; triosephosphate isomerase *tpiB*, AN5908; and glyceraldehyde-3-phosphate dehydrogenase *gpdC*, AN2583), suggesting the incorporation of these carbon sources into other metabolic pathways. For example, the activation of the non-oxidative phase of the pentose phosphate pathway (involved in the biosynthesis of precursors of nucleotides and some amino acids) is supported by the up-regulation of ribose 5-phosphate isomerase (AN5907) and deoxyribose-phosphate aldolase (AN4772). Most metabolic pathways of amino acids were affected by choline supplementation, up-regulating genes involved in the metabolism of cysteine and methionine; aspartate, alanine and asparagine; branched and aromatic amino acids; glutamate, glutamine and proline, among others (Fig. 4, Additional file 1). Excessive supplementation with choline induced accumulation of cyanase [31]; consistent with the up-regulation of the encoding gene (AN7331) observed here (Fig. 4, Table 2). Since cyanide mineralisation is mediated by this enzyme [64], the accumulation of this toxic compound may partially explain how excess choline may result in growth inhibition and activation of stress response in *A. nidulans*.

Major down-regulation on the primary metabolism of *A. nidulans* was provoked by 1-ethyl-3-methylimidazolium chloride, as previously suggested by our proteomic study [31]. Genes involved in glycolysis and TCA cycle were mostly down-regulated, probably a consequence of nutrient limitation (Fig. 4, Additional file 1). This is also strongly sustained by the down-regulation of the majority of the genes involved in the biosynthesis of amino acids. A few exceptions were observed, such as the up-regulation of genes involved in alanine, aspartate and asparagine metabolism, namely asparaginases (*ahtA,* AN0300; AN1891; and AN9195), asparagine synthetase (AN4401) and aspartate aminotransferase (AN1993). This is suggestive of degradation of asparagine into aspartate, followed by incorporation into the central carbon metabolism via oxaloacetate. Up-regulation of genes coding for glycogen debranching enzyme (AN10060) and β-glucosidase (AN10124), involved in the degradation of glycogen and glucans, respectively, points to the use of cellular reserves. The data suggest that carbohydrates originating from these reserves enter glycolysis/gluconeogenesis and are channelled to the pentose phosphate pathway (Fig. 4). 1-Ethyl-3-methylimidazolium chloride induced also the up-regulation of genes involved in the oxidative phase of the pentose phosphate pathway, 6-phosphogluconolactonase (AN0285), 6-phosphogluconate dehydrogenases (AN6135 and AN10233) and ribulose-phosphate 3-epimerase (AN7588) (Fig. 4). A similar effect was noticed before, although more evident in *N. crassa* than in *A. nidulans* [31]. Activation of this pathway leads probably to higher NADPH levels, which plays important roles in antioxidant defence [65].

Both ionic liquids apparently stimulated glutathione biosynthesis, known to play a key role in the stress response in filamentous fungi. This assumption is supported by the up-regulation of its biosynthetic genes, namely gamma-glutamyltranspeptidases genes (*ggtA*, AN10444; and AN5658) and gamma-cysteine synthetase regulatory subunit gene (AN2514) (Fig. 4, Table 2). *Aspergillus nidulans* up-accumulated a glutathione S-transferase in media supplemented with 1-ethyl-3-methylimidazolium chloride [31]. Glutathione S-conjugates are probably formed in the presence of either ionic liquid, shown here by the up-regulation of genes encoding glutathione S-transferases (AN3299 and AN6158) (Fig. 4, Table 2). Accordingly, we also observed that genes coding for efflux pumps (multidrug transporters) belonging to the ATP-binding cassette (ABC) superfamily and the major facilitator superfamily (MFS) were up-regulated (Table 2). MFS multidrug transporters have been reported to participate in detoxification of 1-ethyl-3-methylimidazolium chloride in *Enterobacter lignolyticus* [66]. Activation of both conjugation reactions and efflux pumps is most likely involved in the ionic liquids’ detoxification processes in *A. nidulans*.

Both ionic liquids have possibly triggered the production of acetyl-CoA through pyruvate metabolism (pyruvate decarboxylase *pdcB,* AN8396; aldehyde dehydrogenase *aldA,*AN0554; and acetyl-CoA synthetase *facA,* AN5626) or the degradation of amino acids, such as branched amino acids (Fig. 4). Apart from being channelled to central metabolic pathways, acetyl-CoA is also a key precursor in the synthesis of numerous SMs, *e.g.* elongation of polyketide chains [67]. Also supporting production of SMs, we observed here the up-regulation of 1-aminocyclopropane-1-carboxylate deaminase gene (AN8899) in either ionic liquid media. The encoded enzyme was not found in the mycelial proteome of *A. nidulans* cultures exposed to an ionic liquid, notwithstanding 1-aminocyclopropane-1-carboxylate deaminase accumulated in *N. crassa* grown in similar conditions [31]. Importantly, this enzyme mediates formation of the rare amino acid 1-aminocyclopropane-1-carboxylate found in bioactive SMs classes such as neoefrapeptins and acretocins [68, 69].

### Ionic liquids impact on secondary metabolism of *A. nidulans* – backbone genes

Genes involved in biosynthesis of a particular SM are usually clustered and comprise the backbone gene responsible for biosynthesis of the metabolite core structure and genes coding for additional tailoring enzymes [12, 70]. The backbone gene alone can be responsible for the production of a specific SM (*e.g.* microperfuranone [15] and orsellinic acid [17]), sometimes even when the expression of other clustered genes remains unaltered (*e.g.* penicillin [71]). Overexpression of backbone genes can also result in high production titres of certain SMs (*e.g.* alternariol [72]).

**Fig. 5:**
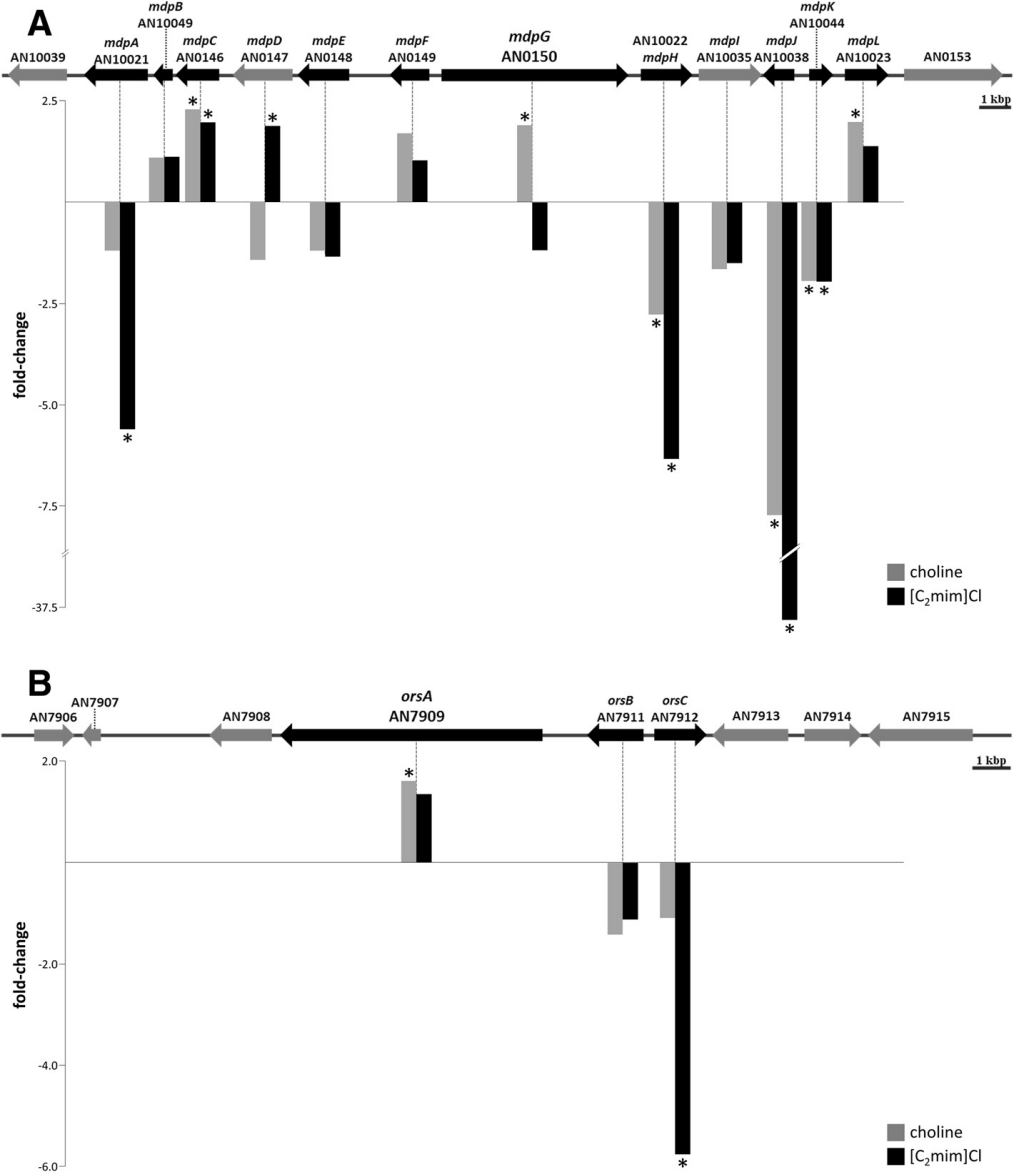
Expression profile of component genes of the monodictyphenone and orsellinic acid clusters under ionic liquid stimuli. Representation of the gene clusters involved in the biosynthesis of monodictyphenone (**a**) and orsellinic acid (**b**) in *Aspergillus nidulans*. Essential and non-essential genes are shown in black and grey (arrows), respectively (adapted from [4, 37]). Below each gene the measured fold-change (FC) in either choline or 1-ethyl-3-methylimidazolium chloride ([C_2_mim]Cl) supplemented media are depicted (microarray data) and, if statistically significant (|FC| ≥ 1.5 and *p*-value ≤ 0.05), indicated with an asterisk (*)

Consistent with the greater diversity of putative compounds found in cultures exposed to either ionic liquid (Table 1, Additional file 3), in total twenty one of the sixty six predicted backbone genes [3] were found up-regulated compared to the control (Table 3 and Additional file 2). Fifteen and nine backbone genes were found up-regulated in choline and 1-ethyl-3-methylimidazolium chloride supplemented media, respectively. This includes *mdpG* in the choline medium and *orsA* in both media. Only three genes coincided between the two conditions, suggesting each ionic liquid induces a specific stimulus. In addition to *mdpG*, the monodictyphenone cluster includes *mdpA* (AN10021) and *mdpE* (AN0148), none of which underwent differential expression in choline media (Fig. 5a). These genes encode transcription factors required for full activation of the monodictyphenone cluster; their deletion usually decreases production titres of monodictyphenone, emodin and derivatives [4]. In addition, *mdpC* and *mdpL* were up-regulated, consistent with monodictyphenone formation. The microarray data support the ability of choline supplement to stimulate formation of monodictyphenone, probably due to up-regulation of *mdpG*, especially since *cclA* expression levels remained unaltered (Additional file 2). De-repression of *mdpG* increased significantly over time in choline supplemented media compared to control (Table 4), probably explaining monodictyphenone identification also on the fifth and tenth days of incubation (Table 1).

**Table 3:** Secondary metabolite synthase genes up-regulated upon ionic liquid stimuli

		Transcriptional profile*			
GeneID	Gene	Choline	[C_2_mim]Cl	Enzyme	Secondary metabolite
AN0150	*mdpG*	**1.90**	−1.18	PKS	monodictyphenone; emodin derivatives [4]
AN0607	*sidC*	**4.78**	**−2.28**	NRPS	ferricrocin (siderophore) [89, 90]
AN1594		1.08	**1.75**	DTS	*ent*-pimara-8(14),15-diene [91]
AN10486		**1.52**	−1.19	NRPS-like	
AN11080	*nptA*	**6.84**	**−12.35**	DMATS	nidulanin A [14]
AN11191		**1.83**	−1.27	PKS	
AN11820		−1.39	**2.00**	NRPS-like	
AN1680		1.20	**1.72**	NRPS-like	
AN2064		1.16	**2.05**	NRPS-like	
AN2547	*easB*	**2.80**	**−2.67**	PKS	emericellamide [7]
AN3230	*pkfA*	1.02	**1.78**	PKS	aspernidine A [11]
AN3396	*micA*	**1.80**	−1.04	NRPS-like	microperfuranone [15]
AN4827		**1.60**	1.13	NRPS-like	
AN5318		**2.39**	**3.20**	NRPS	
AN6236	*sidD*	**1.52**	**−2.80**	NRPS	triacetylfusarinine C (siderophore) [90]
AN6784	*xptA*	−1.53	**1.88**	DMATS	prenyl xanthones [5]
AN6791		**1.67**	1.13	PKS	
AN7071	*pkgA*	**2.37**	**1.89**	PKS	alternariol; isocoumarins [72]
AN12331		**1.86**	**1.56**	PKS-like	
AN7909	*orsA*	**1.61**	1.35	PKS	orsellinic acid; F9775A/B [17]; violaceols [43]
AN9005		**6.81**	**−2.28**	PKS	

Microarray analyses of the backbone genes up-regulated (fold-change, FC) in choline or 1-ethyl-3-methylimidazolium chloride ([C_2_mim]Cl) supplemented media in pair-wise comparison with the control. Values highlighted in bold are statistically significant (|FC| ≥ 1.5 and *p*-value ≤ 0.05)

^*^values highlighted in bold have |FC| ≥ 1.5 and *p*-value ≤ 0.05 in the microarray data. NRPS = non-ribosomal peptide synthase; PKS = polyketide synthase; DMATS = dimethylallyl tryptophan synthase (prenyltransferase); DTS = diterpene synthase

**Table 4:** Time-course analysis of gene expression of orsellinic acid and monodictyphenone synthases and major regulators

			*q*RT-PCR^a^		
Media	GeneID	Gene	5d	10d	15d
Choline	AN7909	*orsA*	2.04	−3.62	1.49
	AN0150	*mdpG*	−57.33	−44.94	−2.66
	AN0807	*laeA*	−10.09	−4.32	−10.00
	AN4562	*rsmA*	−1.64	1.43	2.30
	AN1052	*veA*	−2.91	−1.77	−1.00
[C_2_mim]Cl	AN7909	*orsA*	24.65	1.29	3.42
	AN0150	*mdpG*	−5.54	−74.07	−39.50
	AN0807	*laeA*	−6.93	−13.87	−31.40
	AN4562	*rsmA*	1.94	−2.02	1.09
	AN1052	*veA*	−4.14	−6.85	1.65

*q*RT-PCR analysis of the expression of *orsA* and *mdpG* (backbone genes of orsellinic acid and monodictyphenone, respectively), and *laeA*, *veA* and *rsmA* (secondary metabolism regulatory genes) along the incubation time in choline or 1-ethyl-3-methylimidazolium chloride ([C_2_mim]Cl) supplemented media. Values represent relative gene expression at each culture time in pair-wise comparisons with the control. Expression of each gene was normalised to the expression of the histone protein H3 gene (AN0733)

^a^Fold changes (FCs) in pair-wise comparison with the control

Only *orsA* out of the three genes of the orsellinic acid cluster is necessary for the production of this metabolite [17]. Orsellinic acid detection is consistent with up-regulation of *orsA* in both ionic liquid supplemented media (Table 3), regardless of *orsB* and *orsC* expression values (Fig. 5b). *orsA* underwent major up-regulation during exposure to either ionic liquid, although its expression was transient (Table 4). In the choline supplemented medium, the accumulation profile over time of orsellinic acid parallels *orsA* expression (Table 1). In 1-ethyl-3-methylimidazolium chloride medium, despite *orsA* being more prominently up-regulated on the fifth day of incubation, this SM was detected only at longer incubation times (Table 1). The activities of the histone acetyltransferases EsaA (AN10956) [73] and GcnE (AN3621) [74] were reported to activate expression of penicillin, terrequinone A and sterigmatocystin gene clusters, whereas GcnE influences also *orsA* expression. None of the ionic liquids led to differential expression of *esaA* and *gcnE*, or the backbone genes of penicillin, terrequinone A and sterigmatocystin (Additional file 2). In agreement, penicillin and sterigmatocystin were confirmed to be absent in the culture media (Additional file 3). Putative identification of terrequinone A only occurred in control cultures (data not shown), which may be explained by the major down-regulation of its backbone gene in both ionic liquid media (Additional file 2). None of the identified ion masses matched compounds assigned to the remaining up-regulated backbone genes (Table 3), probably because their abundance is below the detection limit or accumulated intracellularly, or other yet uncharacterised metabolites from the cluster were produced instead.

### Ionic liquids impact on secondary metabolism of *A. nidulans* – regulatory genes

A complex regulatory network governs the co-regulation of the clustered SM biosynthetic genes [75], involving hierarchical levels of transcriptional regulatory elements. These can be either pathway-specific (*e.g.* AflR in the sterigmatocystin/aflatoxin gene cluster [76]) or broad domain transcription factors (*e.g.* the Velvet complex), and proteins responsive to general environmental factors that are also implicated in cluster activation (*e.g.* CreA, PacC and AreA) [77]. Global regulators of secondary metabolism in *Aspergillus* spp. include the well-studied VelB/VeA/LaeA transcriptional complex (*i.e.* Velvet complex) that links secondary metabolism with fungal development [75, 78]. In the dark – similar to culture conditions used here – the Velvet complex controls the activity of LaeA (methyltransferase-domain nuclear protein), which in turn controls the expression of several SM gene clusters [79, 80]. The impact of LaeA on the regulation of secondary metabolism has been well studied in *A. fumigatus* (the *laeA* deletion mutant shows repression of 13 of 22 SM biosynthetic clusters) [81], but is also known to impact other fungi (*e.g. Fusarium verticillioides* [82]). It controls, in general, gene clusters positioned at the telomere proximal region of the chromosomes [83]. Among the backbone genes found up-regulated here only few are located proximal to the telomere, consistent with studies implicating other secondary metabolism regulatory elements apart from LaeA [15].

LaeA (AN0807) is required for biosynthesis of sterigmatocystin and penicillin [78]. However, sterigmatocystin biosynthesis can be restored in *laeA* or *veA* deletion strains by the up-regulation of *rsmA* (*r*emediation of *s*econdary *m*etabolism *A*, AN4562) [84] or the deletion of *mtfA* (*m*aster *t*ranscription *f*actor *A*, AN8741) [85], respectively. Our microarray data showed that both ionic liquids led to down-regulation of *laeA*, whereas *veA* (AN1052) was down-regulated only in 1-ethyl-3-methylimidazolium chloride supplemented medium and *velB* (AN0363) was not differentially expressed in either condition (Table 5). *q*RT-PCR analyses of expression levels of *laeA* and *veA* over the incubation period (up to fifteen days) confirmed their down-regulation (Table 4). In choline supplemented medium, despite the up-regulation of *rsmA* over time (Table 4) sterigmatocystin accumulation was not validated (Additional file 3). In addition, up-regulation of *mtfA* in 1-ethyl-3-methylimidazolium chloride medium likely led to the down-regulation of sterigmatocystin and terrequinone A backbone genes (Additional file 2), hence the absence of these SMs in the cultures. Penicillin accumulation in the culture was not observed, consistent with the down-regulation of *laeA*, which is required for its biosynthesis [78].

**Table 5:** *q*RT-PCR analysis of selected genes encoding secondary metabolism components, either biosynthesis or regulation

		Choline		[C_2_mim]Cl	
GeneID	Gene	*q*RT-PCR	Microarray^*^	*q*RT-PCR	Microarray^*^
*Backbone genes*					
AN7909	*orsA*	1.67	**1.61**	2.57	1.35
AN0150	*mdpG*	−3.62	**1.90**	−12.50	−1.18
AN8383	*ausA*	−6.00	−1.30	−3.04	**−2.26**
AN2547	*easB*	2.08	**2.80**	−4.92	**−2.67**
AN0607	*sidC*	3.06	**4.78**	−4.57	**−2.28**
*Transcriptional regulators*					
AN0807	*laeA*	−6.75	**−6.71**	−25.32	**−17.55**
AN1052	*veA*	−1.08	−1.23	−1.23	**−1.52**
AN0363	*velB*	−1.91	−1.16	1.43	1.45
AN4819	*fluG*	−1.26	−1.12	−1.89	−1.45
AN4562	*rsmA*	−1.21	1.12	−1.66	−1.18
AN1905	*hepA*	−1.06	1.18	1.23	**1.70**
AN8042	*hdaA*	1.39	1.04	1.14	−1.18
AN3621	*gcnE*	1.14	−1.34	1.26	−1.30

Values represent the relative expression of selected genes in pair-wise comparisons with the control, after fifteen days of incubation. Expression of each gene was normalised to the expression of the histone protein H3 gene (AN0733). Corresponding microarray data are shown for comparison

*values highlighted in bold have |FC| **≥** 1.5 and *p*-value **≤** 0.05 in the microarray data

Additional genes involved in histone modifications can also impact SM production. Deletion of the histone deacetylase gene *hdaA* (AN8042) for example induces the production of sterigmatocystin and penicillin [86] and high levels of the heterochromatin protein HepA (AN1905) provoke opposite effects [87]. *hdaA* was not differentially expressed but *hepA* was up-regulated in 1-ethyl-3-methylimidazolium chloride supplemented medium (Table 5), probably contributing to the repression of sterigmatocystin and penicillin biosynthesis in this medium.

A set of thirteen genes involved in secondary metabolism, either coding for SM synthases (*mdpG, orsA*, *sidC*, *easB* and *ausA*), chromatin remodelling enzymes (*hepA*, *hdaA* and *gcnE*) or regulatory proteins, including those discussed above (*laeA*, *rsmA, veA* and *velB*), as well as *fluG*, were selected to validate the microarray data by *q*RT-PCR (Table 5). With few exceptions the majority of the analysed genes displayed an expression profile similar to that detected by microarray analyses.

### Analysis of the biological activity of *A. nidulans* metabolite extracts after ionic liquid stimuli

**Fig. 6:**
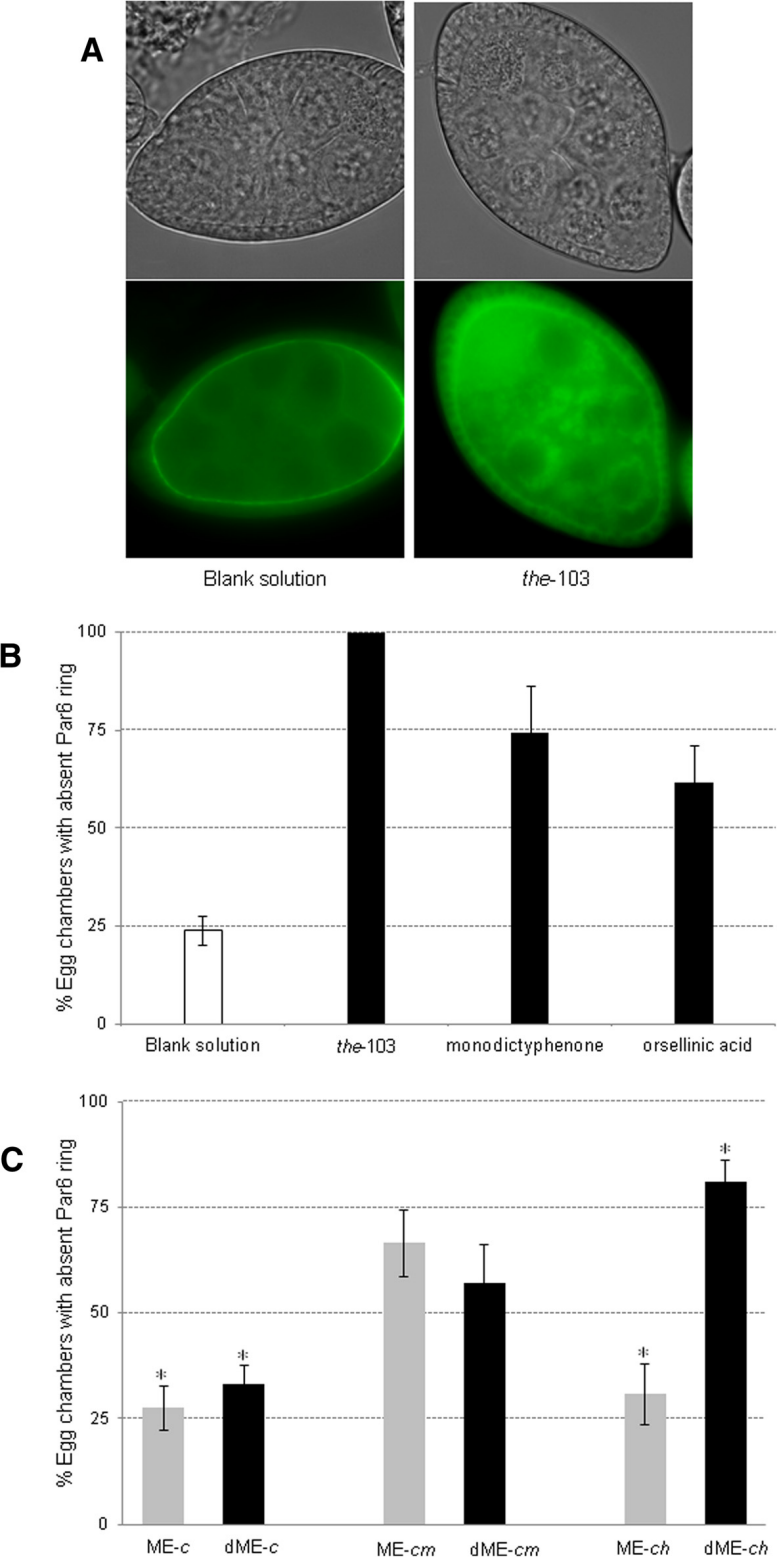
*Ex vivo the*LiTE™ assay of *Aspergillus nidulans* metabolite extracts from ionic liquid supplemented media. Microscope image of *Drosophila* egg chambers showing presence/absence of the polarity marker protein Par6 when exposed to solvent only solution (0.6 % DMSO, *i.e*. blank) and *the*-103, respectively (**a**); % of egg chambers showing Par6 ring impairment in the presence of 24 μg/mL monodictyphenone, orsellinic acid, *the*-103 (the blank is also shown) (**b**) or metabolite extracts collected at incubation day seven in the control medium (ME-*c*) or either choline (ME-*ch*) or 1-ethyl-3-methylimidazolium chloride ([C_2_mim]Cl) (ME-*cm*) supplemented media, before (grey bars) or after dereplication of its polar fraction (dME) (black bars) (**c**). The asterisk denotes a significant difference (*p*-value ≤ 0.05) of each treatment compared to the control

To screen the presence of compounds displaying anti-carcinoma potential in the metabolite extracts of *A. nidulans* an *ex vivo* polarity modulation assay (*the*LiTE™) was used. The positive control (30 μM of *the*-103 compound) affected 100 % of the *Drosophila* eggs, whereas the blank (*i.e.* 0.6 % v/v DMSO which is the solution used to solubilise the metabolite extracts) is devoid of any significant activity (Fig. 6a-b). The capacity of a pure compound to impair the fluorescent ring of the polarity marker protein Par6 in more than *ca.* 50 % of the eggs is defined as the empirical cut off for a positive result. When dealing with complex mixtures, much weaker activities should be regarded as significant, especially if a de-replicated fraction shows substantially higher activity. Under standard cultivation conditions (control), metabolite extracts (crude and its corresponding polar fraction) were virtually devoid of activity (Fig. 6c); both ionic liquids induced the biosynthesis of compounds carrying anti-carcinoma potential. Similar activities were, in general, measured for the metabolite extracts derived in either ionic liquid media at the seventh or the fifteenth day of incubation, which were much stronger that those measured at the second day (data not shown). For example, crude metabolite extracts (24 μg/mL) from 1-ethyl-3-methylimidazolium chloride and choline supplemented media, at the seventh day of incubation, showed high to moderate activity (67 and 31 %, respectively); their polar fractions (de-replicated metabolite extracts, dME) showed similar or much greater activity (57 and 81 %, respectively) (Fig. 6c). The detected activities cannot be related to any vestigial amounts of either ionic liquid, because these compounds *per se* lack activity (data not shown). We verified also that 24 μg/mL of monodictyphenone (83 μM) or orsellinic acid (129 μM) affected 75 or 62 % of the eggs, respectively (Fig. 6b). Both SMs, primarily monodictyphenone, may contribute to the polarity modulating activity of the metabolite extracts (either crude or de-replicated); additional active components probably remain obscured. The future challenge is to identify, among the compounds specific to each ionic liquid, those that carry intrinsic anti-carcinoma activity.

## Conclusions

Transcriptional profiling was used for the first time to evaluate ionic liquids broad impacts on both primary and secondary metabolism of *A. nidulans*. Primary metabolism was up-regulated by choline, but down-regulated by 1-ethyl-3-methylimidazolium chloride. Choline could be used as a source of carbon and nitrogen probably via the glycine, serine and threonine metabolic pathway, and incorporated into the central carbon metabolism. On the contrary, the recalcitrant 1-ethyl-3-methylimidazolium chloride induced the use of cellular reserves and autophagy. Both ionic liquids induced detoxification mechanisms (*viz*. multidrug transporters and glutathione S-conjugates) probably to eliminate the toxic cations or toxic intermediates (*e.g.* cyanide). Despite strongly contrasting effects on primary metabolism either ionic liquid apparently stimulated production of acetyl-CoA, a key precursor to numerous SMs, as well as the production of non proteinogenic amino acids known as building blocks of bioactive classes of SMs. Differential analyses of the fungal metabolome allowed discovery of numerous putative SMs, including gentisic acid and caffeic acid here reported for the first time in *A. nidulans.* Concurrently with numerous differentially formed compounds, multiple genes encoding SM biosynthetic enzymes were up-regulated. Each ionic liquid stimulus activated a specific set of backbone genes, including uncharacterised ones. In addition, growth media supplementation with choline led, for the first time, to monodictyphenone accumulation in a wild type strain of *A. nidulans*. Importantly, we observed here that the complex mixtures of the formed metabolites camouflage compounds with anti-carcinoma potential. This study should inspire the search of novel bioactive fungal SMs biosynthesised under an ionic liquid stimulus.

Ionic liquids impact on eukaryotic organisms constitutes a fundamental cue for conscious development of this field. This study constitutes a step forward to prior proteomic analyses of ionic liquids’ impacts in primary metabolism, providing a more detailed analysis and expanding initial findings to secondary metabolism. We believe it illustrates the potential of ionic liquids to induce metabolic alterations and stress responses in eukaryotic organisms. In particular, we showed that they can be used to further resolve the diversity of natural compounds, guiding discovery of fungal metabolites with clinical potential. Further studies are necessary to elucidate the capacity of the ionic components to specifically modulate defined response regulators of metabolism and development in fungi. Ionic liquids formation during the confrontation between two ant species has been recently demonstrated, probably as a defence mechanism [88]. The likelihood of natural ionic liquids creates a new paradigm – they are not exclusively man-made chemicals – and supplies a new boost of interest in their research. Unforeseen possibilities have been revealed and future exploration promises to be exciting.

### Availability of supporting data

Data sets supporting the results of this article are included within the article (and its additional files).

## Additional files

Additional file 1: Microarray additional details, experimental data and validation. (PDF 649 kb) Additional file 2: Microarray raw data and list of differentially expressed genes. (XLSX 3421 kb) Additional file 3: List of all the differential compounds identified in the culture extracts. (XLSX 107 kb)

## Abbreviations

FC: fold change
MIPS: Munich Information Center for Protein Sequences
*q*RT-PCR: quantitative real-time PCR
SMs: secondary metabolites
TCA: tricarboxylic acid cycle
UHPLC-ESI-HRMS: ultra-high performance liquid chromatography-electrospray ionisation-high resolution mass spectrometry
