# Supplementary material for: Transcriptomic and metabolomic profiling of ionic liquid stimuli unveils enhanced secondary metabolism in Aspergillus nidulans
Source: BMC Genomics. 2016 Apr 12;17:284. doi: 10.1186/s12864-016-2577-6 (PMC4830055; doi:10.1186/s12864-016-2577-6)
Supplement: Additional file 1: — Microarray additional details, experimental data and validation. (PDF 649 kb) [file 12864_2016_2577_MOESM1_ESM.pdf]

Additional file 1

Transcriptomic and metabolomic profiling of ionic liquid stimuli unveils enhanced secondary metabolism in *Aspergillus nidulans*

Paula C. Alves[a,#], Diego O. Hartmann[a, #], Oscar Núñez[b,c], Isabel Martins[a], Teresa L. Gomes[d], Helga Garcia[a], Maria Teresa Galceran[b], Richard Hampson[d], Jörg D. Becker[e] and Cristina Silva Pereira[a*]

[a] Instituto de Tecnologia Química e Biológica António Xavier, Universidade Nova de Lisboa, Av. da República, 2780-157 Oeiras, Portugal

[b] Department of Analytical Chemistry, University of Barcelona, Diagonal 645, E-08028 Barcelona, Spain

[c] Serra Hunter Fellow, Generalitat de Catalunya, Spain

[d] Thelial Technologies S.A., Parque Tecnológico de Cantanhede, Nucleo 04 Lote 3, 3060-197 Cantanhede, Portugal

[e] Instituto Gulbenkian de Ciência, Rua da Quinta Grande 6, 2780-156, Oeiras, Portugal

[#] Equally contributing authors

* Corresponding author:

Cristina Silva Pereira

Instituto de Tecnologia Química e Biológica, Universidade Nova de Lisboa, Av. da República, EAN, 2781-901, Oeiras Portugal

Tel.+351 211157786

E-mail: spereira@itqb.unl.pt

Contents: Microarray additional details, experimental data and validation

1. Details on the mapping strategy 3

1.1. Custom FungiANC microarray design

1.2. Custom FungiANC microarray re-annotation

2. Overview of the microarray data 4

3. List of the designed qRT-PCR oligonucleotides used in the validation of the microarray data 5

4. Functional Categories altered in *Aspergillus nidulans* after exposure to choline and 1-ethyl-3-methylimidazolium chloride. 6

5. Genes of primary metabolism differentially expressed after ionic liquid stimuli............................7

1. Details on the mapping strategy

1.1. Custom FungiANC microarray design

The nucleotide sequences used for microarray design were from *Aspergillus nidulans* FGSC A4 (v.7, *Aspergillus* Comparative Sequencing Project) and *Neurospora crassa* FGSC 2489 (v.3, *Neurospora crassa* Sequencing Project) genomes retrieved from Broad Institute of Harvard and MIT database (http://www.broadinstitute.org, May 2010). The parameters chosen for the custom array design were as follows: 11-µm feature size, 49-5241 array format, eukaryotic antisense target type, perfect-match probes only, probe selection within the 600 bp from the 3' end and eight probes *per* sequence as the minimum acceptable. The types of probe sets represented are: 19,839 unique; 77 gene (_a); 91 identical (_s) and 58 mixed (_x). *Aspergillus nidulans* FGSC A4 genes are represented by 10,252 unique probes; 75 gene (_a); 85 identical (_s) and 45 mixed (_x). *Neurospora crassa* FGSC 2489 genes are represented by 9,545 unique probes; 2 gene (_a); 3 identical (_s) and 13 mixed (_x).

Probe gene coverage was calculated as 98 % and 99.8 % for *A. nidulans* and *N. crassa,* respectively.

1.2. Custom FungiANC microarray re-annotation

In order to update the annotation of our custom microarray, the transcript sequences were aligned against all chip probe sequences, based on NCBI BLASTN searches (e-value = 10).

Only the alignments with minimal length of 17 bases long were kept and designated as "matching probes". The existence of at least 6 matching probes to the same transcript was considered the "matching probeset" of that transcript. At last, it was assigned a "Confidence Level" of the mapping according to the total number of mismatch bases considering the total number of probes in the probeset. This way, the levels established were "Certain" (≤ 0, *i.e.* all perfectly aligned), "Highly Probable" (≤ 25), "Probable" (≤ 50), "Likely" (≤ 75) and "Possible" (other number of mismatch bases).

2. Overview of the microarray data. A) Principal Component Analysis. B) Volcano plots: plotting of the statistical significance of the microarray data ($-\log_{10}$ adjusted *p*-value) against the fold-change ($\log_2$ ratio) for *Aspergillus nidulans* in the different tested media. Significant genes have positive ($-\log_{10}$) adjusted *p*-values and they can show different levels of fold-changes. C) Venn diagrams: number of differentially expressed genes in *Aspergillus nidulans* in the different tested media versus the control.

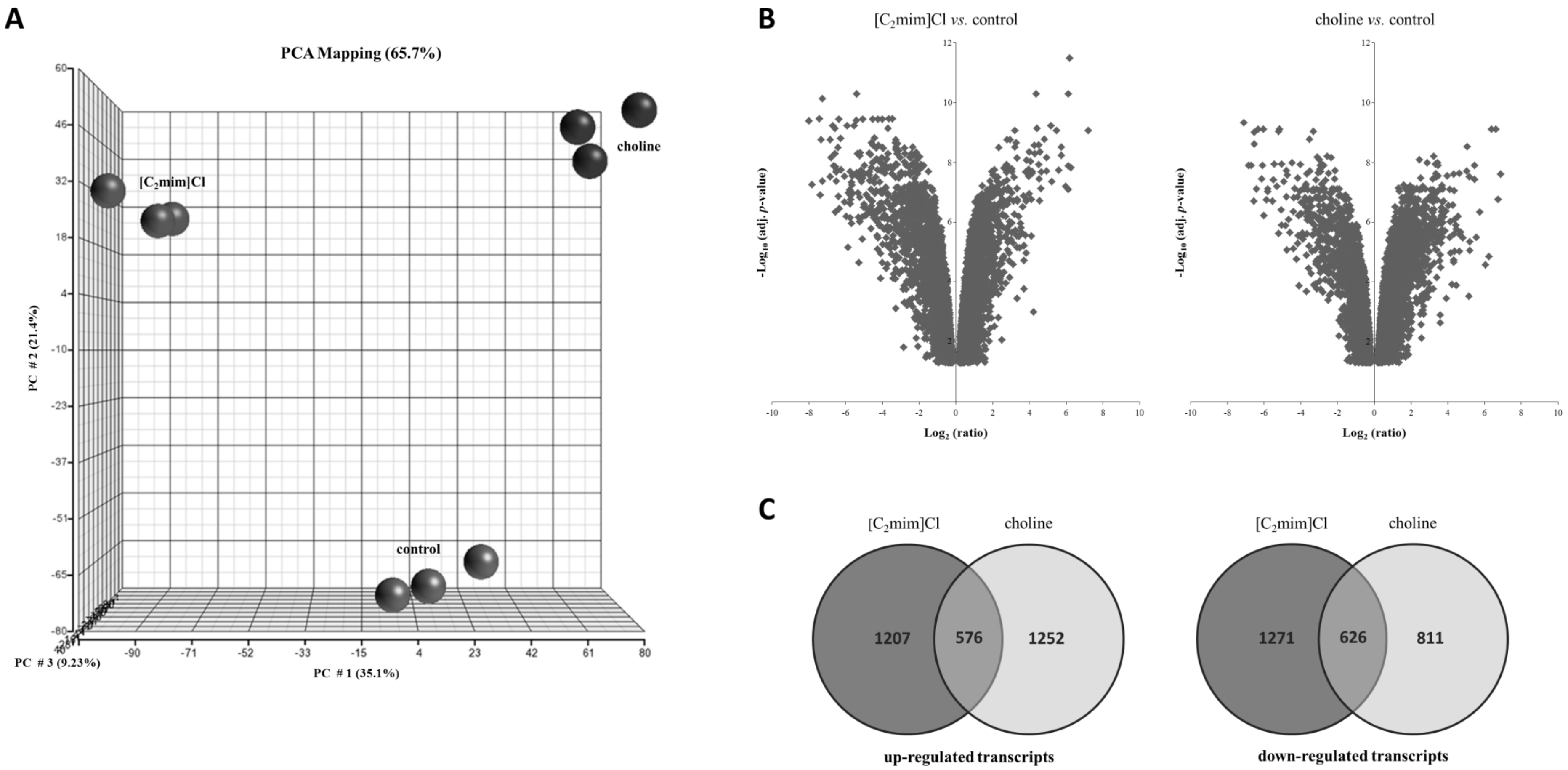

3. List of the designed *q*RT-PCR oligonucleotides used in the validation of the microarray data. Due to its constant expression in all conditions, the histone protein H3 gene (AN0733) was selected as internal control.

GeneID	Gene	Forward oligonucleotide (5′ to 3′)	Reverse oligonucleotide (5′ to 3′)
AN1052	*veA*	TCAGAGCTCCCATCGACCA	GCCGGTCATCATGACCGAA
AN0363	*velB*	CCCAAGCCCAGTACTACCAA	CGGTATGCACTGGCACTCA
AN0807	*laeA*	ATGGGTTGCGGTAGTGTCA	CAATGCCGTTCCATCTAGTGA
AN4562	*rsmA*	CCTCCGCGCTACAACACA	AGTCCGTAGGAGAGTAGACCA
AN4819	*fluG*	AGAACCGTGAGGCGCCTA	GTATATCCCGCAGCCAGGAA
AN8042	*hdaA*	CATCCAGGGAAGGACTTAGTGA	TTGGGAGCGGCCTTGGTA
AN8383	*ausA*	TACAACCCTCCACCCAGCA	TCGGGCCCTGTAGTTGCA
AN1905	*hepA*	GGCGAAGATAAAGGAAAGCGTA	CTTCTTGCCGTTATTCCAGTGA
AN3621	*gcnE*	AATGGATCTGTCGACGATGGA	GTTCGCGCTCTTGGCGTA
AN2547	*easB*	ATCGCCTCCGACGACGAA	GTGTTGCCGCTGATAGGAGA
AN0150	*mdpG*	CGTCAAGATGATTCAGACCGAA	GCCGTGAAGAACCTATTAAGCA
AN7909	*orsA*	CACCATTTTCCTGCGCACA	GAGGATGATAAGGGCCTGGA
AN0607	*sidC*	CATTCAGAGATCCCTCGCAGA	AAGTGCTGTCCTGCATCCAA
AN0733	*H3*	TGAGATCCGTCGCTACCAGA	AACGGACTCCTGGAGAGCA

4. Functional Categories altered in *Aspergillus nidulans* after exposure to choline and 1-ethyl-3-methylimidazolium chloride ([C_2mim]Cl).

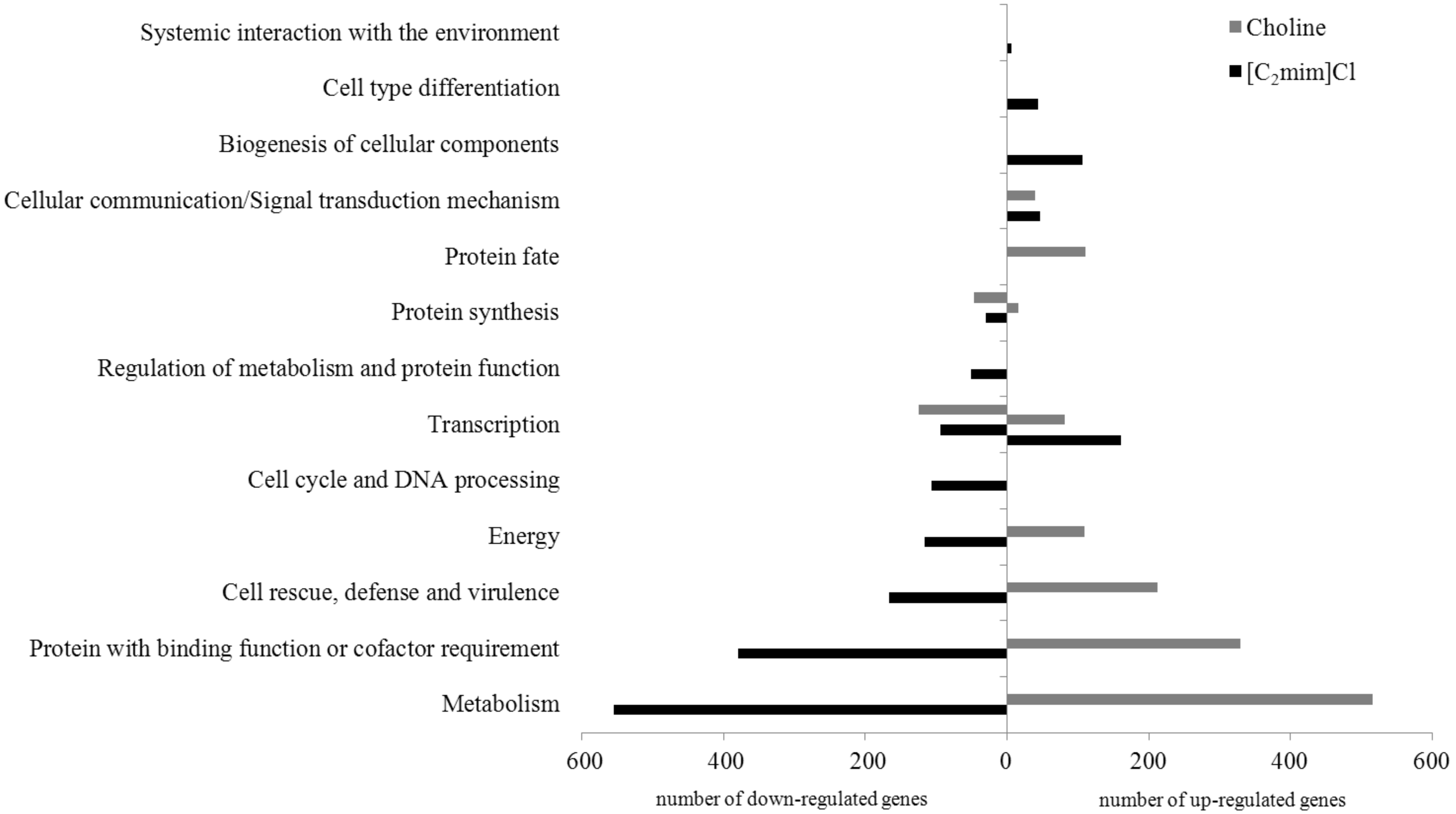

5. Genes of primary metabolism differentially expressed after ionic liquid stimuli. Microarray analyses (fold-change, FC) in choline or 1-ethyl-3-methylimidazolium chloride ([C_2mim]Cl) supplemented media in pair-wise comparisons with the control. Values highlighted in bold are statistically significant (|FC| ≥ 1.5 and *p*-value ≤ 0.05).

		**Transcriptional profile***		
**GeneID**	**Gene**	**Choline**	**[C_2mim]Cl**	**Description**
CARBOHYDRATE METABOLISM				
Glycolysis and gluconeogenesis				
AN2867	*pgmB*	**-1.82**	1.19	phosphoglucomutase
AN4591		**1.95**	**-1.61**	phosphoglucomutase
AN6037	*swoM*	**-1.70**	**1.53**	glucose 6-phosphate isomerase
AN1888		-1.03	**-1.70**	fructose-bisphosphate aldolase
AN2334		**3.39**	**1.81**	fructose-bisphosphate aldolase
AN6900	*tpiA*	**-1.78**	1.14	triosephosphate isomerase
AN5908	*tpiB*	**1.73**	**-2.08**	triosephosphate isomerase
AN2583	*gpdC*	**3.32**	**7.66**	glyceraldehyde-3-phosphate dehydrogenase
AN1873		1.57	**1.81**	phosphoglycerate mutase
AN4983		-1.23	**2.15**	phosphoglycerate mutase
AN8720		1.06	**2.35**	phosphoglycerate mutase
AN5746	*acuN*	1.43	**-1.56**	enolase
AN5210	*pkiA*	-1.39	**-1.68**	pyruvate kinase
AN5843	*pdkA*	**-5.51**	**-10.15**	phosphoenolpyruvate synthase
Pyruvate metabolism				
AN8396	*pdcB*	**2.68**	**-2.65**	pyruvate decarboxylase
AN0554	*aldA*	**1.53**	**-2.21**	aldehyde dehydrogenase
AN5626	*facA*	**1.55**	**-3.26**	acetyl-CoA synthetase
TCA cycle and glyoxylate shunt				
AN2435	*aclA*	1.35	**2.16**	ATP citrate synthase
AN2436	*aclB*	1.19	**1.81**	ATP citrate synthase
AN10973		**-9.47**	**-8.30**	citrate synthase
AN5300		-1.29	**-1.67**	aconitate hydratase
AN3894		**2.17**	1.35	aconitate hydratase
AN7000		**1.73**	1.11	succinate-CoA ligase
AN0896		**1.97**	**-1.93**	succinate dehydrogenase
AN2332		**1.68**	1.13	succinate dehydrogenase
AN2916		**1.86**	**-2.41**	succinate dehydrogenase
AN8707		**1.71**	**-3.89**	fumarate hydratase
AN5634	*acuD*	**2.49**	-1.42	isocitrate lyase
AN6653	*acuE*	**2.30**	-1.00	malate synthase
AN6717	*mdhA*	**1.91**	-1.44	malate dehydrogenase
AN6499	*mdhC*	1.45	**-2.38**	malate dehydrogenase
AN6168	*maeA*	**11.90**	**-3.11**	malate dehydrogenase
AN6933	*maeB*	**-2.04**	**2.39**	malate dehydrogenase
AN1918	*acuF*	**1.85**	-1.11	phosphoenolpyruvate carboxykinase
Pentose phosphate pathway				
AN2981	*gsdA*	**-1.56**	1.30	glucose 6-phosphate 1-dehydrogenase
AN0285		1.24	**1.82**	6-phosphogluconolactonase
AN3954		**-1.54**	1.19	6-phosphogluconate dehydrogenase
AN6135		**2.57**	**1.61**	6-phosphogluconate dehydrogenase
AN10233		**-4.89**	**4.99**	6-phosphogluconate dehydrogenase
AN10783		1.30	**-2.69**	6-phosphogluconate dehydrogenase
AN5907		**2.31**	-1.23	ribose 5-phosphate isomerase
AN7588		-1.02	**2.01**	ribulose-phosphate 3-epimerase
AN4913	*phk*	**-2.63**	**3.45**	phosphoketolase
AN0240	*pppA*	**-1.53**	1.30	transaldolase

GeneID	Gene	Transcriptional profile* Choline	[C_2mim]Cl	Description
AN1965		**-1.64**	1.31	phosphoribosyl diphosphate synthase
AN7995		1.03	**-2.29**	ribokinase
AN4772		**4.69**	-1.19	deoxyribose-phosphate aldolase
Others				
AN10060		-1.14	**2.07**	glycogen debranching enzyme, alpha-amylase
AN10124		1.48	**2.34**	β-glucosidase
AN0712	*bglB*	**-1.55**	-1.42	β-glucosidase
AN2217	*bxlC*	**-1.74**	1.34	β-glucosidase
AN2227	*bglI*	**-2.67**	1.22	β-glucosidase
AMINO ACIDS METABOLISM				
Glutamate, glutamine and proline metabolism				
AN4376	*gdhA*	**2.15**	1.42	NADP-linked glutamate dehydrogenase
AN7451	*gdhB*	1.03	**-2.25**	NAD dependent glutamate dehydrogenase
AN5134	*gltA*	1.00	**-1.54**	glutamate synthase
AN5447		**6.71**	**-2.48**	glutamate decarboxylase
AN7278		**-9.96**	**-17.03**	glutamate decarboxylase
AN2248	*gatA*	**1.98**	**-2.32**	gamma-amino-n-butyrate transaminase
AN1585		1.12	**1.87**	succinate-semialdehyde dehydrogenase
AN3829		**-2.58**	**-1.58**	succinate-semialdehyde dehydrogenase
AN4820		**2.28**	-1.48	succinate-semialdehyde dehydrogenase
AN7315		**1.50**	-1.20	succinate semialdehyde dehydrogenase
AN1733		-1.13	**-2.40**	delta-1-pyrroline-5-carboxylate dehydrogenase
AN6022		-1.08	**1.60**	delta-1-pyrroline-5-carboxylate dehydrogenase
AN7387	*pcrA*	**3.33**	-1.26	pyrroline-5-carboxylate reductase
AN1731	*prnD*	**-4.96**	**-2.88**	proline oxidase
AN5817		1.00	**1.58**	glutamate 5-kinase
AN5799	*proA*	**1.73**	**2.25**	glutamate semialdehyde dehydrogenase
Arginine metabolism				
AN1810	*otaA*	-1.12	**-1.63**	ornithine aminotransferase
AN5867	*ornD*	**-2.08**	-1.06	acetylglutamate synthase
AN8770		**2.41**	-1.33	acetylglutamate kinase
AN5749		**-1.60**	**-1.90**	peptidase
AN2914	*arg1*	-1.47	**-1.86**	argininosuccinate lyase
AN10079	*ureB*	1.08	**-1.70**	urease
Lysine metabolism				
AN1990	*lysD*	**-1.57**	1.27	homocitrate synthase
AN3894		**2.17**	1.35	aconitate hydratase
AN5206	*lysB*	1.49	**-1.50**	isocitrate dehydrogenase
AN5601		1.13	**-1.62**	saccharopine dehydrogenase
Histidine metabolism				
AN3748		1.02	**-1.78**	ATP phosphoribosyltransferase
AN0797		-1.21	**-1.90**	phosphoribosyl-AMP cyclohydrolase
AN0717		-1.19	**-1.94**	histidinol-phosphate aminotransferase
AN7044		1.47	**-2.05**	histidinol phosphatase
AN2723		**3.54**	**1.71**	histidinol dehydrogenase
Tyrosine, phenylalanine and tryptophan				
AN1673		**2.50**	**-2.00**	phospho-2-dehydro-3-deoxyheptonate aldolase
AN5701	*aroF*	**1.81**	**-1.72**	3-deoxy-D-arabino-heptulosonate 7-phosphate synthase
AN0708	*aromA*	**-1.56**	1.11	pentafunctional AROM polypeptide
AN3695		**1.60**	-1.35	anthranilate synthase
AN3634		1.17	**-2.89**	anthranilate phosphoribosyltransferase
AN6866	*aroC*	-1.48	**-1.51**	chorismate mutase
AN6338		**1.69**	**-2.78**	aromatic aminotransferase
Glycine, serine and threonine metabolism				
AN1430		**2.51**	**-1.72**	betaine aldehyde dehydrogenase
AN8654		**20.35**	**-5.15**	dimethylglycine oxidase

GeneID	Gene	Transcriptional profile* Choline	$[C_2mim]Cl$	Description
AN1342		**4.99**	-1.31	aminotransferase
AN10745		**2.46**	-1.22	serine hydroxymethyltransferase
AN3058		**-2.31**	-1.19	serine hydroxymethyltransferase
AN1198		**5.00**	**-2.15**	glycine cleavage system T protein
AN3866		**118.88**	1.23	L-serine dehydratase
AN7564		**-1.87**	**-3.13**	L-allothreonine aldolase
AN8843		-1.45	**-1.96**	homoserine kinase
AN2525		**1.54**	-1.34	pyridoxal-phosphate dependent enzyme
Cysteine and methionine metabolism				
AN0875		-1.15	**-25.05**	homoserine acetyltransferase
AN5820	*mecA*	**1.97**	**-4.91**	cystathionine beta-synthase
AN3456		-1.30	**-1.56**	cystathionine gamma-synthase
AN1446	*mecB*	**2.34**	**2.40**	cystathionine gamma-lyase
AN4443	*metH*	**-1.72**	**-1.83**	methionine synthase
Aspartate and asparagine and alanine metabolism				
AN1993		**2.87**	**1.66**	aspartate aminotransferase
AN6048		**3.90**	**-1.50**	aspartate transaminase
AN8709		**2.32**	-1.11	aspartate transaminase
AN0300	*ahtA*	**1.94**	**1.52**	L-asparaginase
AN1891		-1.16	**4.04**	asparaginase
AN9195		1.46	**2.01**	L-asparaginase II
AN4401		1.02	**1.62**	asparagine synthetase
AN8754		**2.07**	-1.02	asparagine synthase
AN1342		**4.99**	-1.31	aminotransferase
AN5193		**1.64**	1.16	aspartate aminotransferase
Valine, leucine and isoleucine metabolism				
AN4058		-1.43	**-7.94**	dihydroxy-acid dehydratase
AN5138		1.38	**-1.77**	dihydroxy-acid dehydratase
AN6346		-1.04	**-2.07**	dihydroxy-acid dehydratase
AN7358		**1.92**	**4.11**	dihydroxy-acid dehydratase
AN0840		1.19	**-1.51**	2-isopropylmalate synthase
AN5886	*luA*	**1.59**	1.41	3-isopropylmalate dehydratase
AN2793	*leu2B*	**1.85**	**-1.64**	3-isopropylmalate dehydrogenase
AN4323		1.46	**-2.13**	branched-chain amino acid aminotransferase
AN5957		**1.55**	**-1.57**	branched-chain amino acid aminotransferase
AN7876		**2.46**	-1.04	branched-chain amino acid aminotransferase
AN7878		**-1.61**	**-1.83**	branched-chain amino acid aminotransferase
AN5916	*echA*	**1.56**	**-2.91**	enoyl-CoA hydratase/isomerase
AN0574		1.44	**-2.59**	3-oxoacyl-(acyl-carrier-protein) reductase
AN10512	*mthA*	1.43	**-2.71**	acetyl-CoA-acetyltransferase
AN4688	*ivdA*	**2.24**	**-4.73**	isovaleryl-coenzyme A dehydrogenase
AN4687	*mccD*	**1.95**	**-3.48**	3-methylcrotonyl-CoA carboxylase
AN2896		1.46	**-1.89**	mitochondrial methylglutaconyl-CoA hydratase
AN5273	*hlyA*	-1.15	**1.86**	hydroxymethylglutaryl-CoA lyase
AN9131		**-1.68**	-1.19	hydroxymethylglutaryl-CoA lyase
AN10797		1.39	**-2.42**	hydroxymethylglutaryl-CoA lyase
AN5669		**2.50**	**-2.81**	succinyl-CoA:3-ketoacid-CoA transferase
AN8559		**1.83**	**-3.23**	2-oxoisovalerate dehydrogenase
AN0593		**-2.61**	**2.01**	3-hydroxyisobutyrate dehydrogenase
AN3591		**3.36**	**-3.37**	methylmalonate-semialdehyde dehydrogenase
AN10656		-1.19	**2.77**	propionyl-CoA carboxylase
Other				
AN8899		**9.30**	**1.67**	1-aminocyclopropane-1-carboxylate deaminase

*values highlighted in bold have |FC| ≥ 1.5 and *p*-value ≤ 0.05 in the microarray data.
